# A novel DNA methylation-related gene signature for the prediction of overall survival and immune characteristics of ovarian cancer patients

**DOI:** 10.1186/s13048-023-01142-0

**Published:** 2023-03-29

**Authors:** Sixue Wang, Jie Fu, Xiaoling Fang

**Affiliations:** 1 Department of Obstetrics and Gynecology, The Second Xiangya Hospital of Central South University, Changsha, 410011 Hunan China; 2 Department of General Surgery, The Second Xiangya Hospital of Central South University, Changsha, 410011 Hunan China

**Keywords:** DNA methylation, Risk score, Prognosis, Immunotherapy, Ovarian cancer

## Abstract

**Background:**

Ovarian cancer (OC) is one of the most life-threatening cancers affecting women worldwide. Recent studies have shown that the DNA methylation state can be used in the diagnosis, treatment and prognosis prediction of diseases. Meanwhile, it has been reported that the DNA methylation state can affect the function of immune cells. However, whether DNA methylation-related genes can be used for prognosis and immune response prediction in OC remains unclear.

**Methods:**

In this study, DNA methylation-related genes in OC were identified by an integrated analysis of DNA methylation and transcriptome data. Prognostic values of the DNA methylation-related genes were investigated through least absolute shrinkage and selection operator (LASSO) and Cox progression analyses. Immune characteristics were investigated by CIBERSORT, correlation analysis and weighted gene co-expression network analysis (WGCNA).

**Results:**

Twelve prognostic genes (CA2, CD3G, HABP2, KCTD14, PI3, SERPINB5, SLAMF7, SLC9A2, STC2, TBP, TREML2 and TRIM27) were identified and a risk score signature and a nomogram based on prognostic genes and clinicopathological features were constructed for the survival prediction of OC patients in the training and two validation cohorts. Subsequently, the differences in the immune landscape between the high- and low-risk score groups were systematically investigated.

**Conclusions:**

Taken together, our study explored a novel efficient risk score signature and a nomogram for the survival prediction of OC patients. In addition, the differences of the immune characteristics between the two risk groups were clarified preliminarily, which will guide the further exploration of synergistic targets to improve the efficacy of immunotherapy in OC patients.

**Supplementary Information:**

The online version contains supplementary material available at 10.1186/s13048-023-01142-0.

## Introduction

Ovarian cancer (OC) is one of the most lethal malignant tumors affecting women. The incidence rate is third among gynecologic cancers, but the mortality rate is the highest in women around the world [1]. At present, the major treatment of OC is to add maintenance treatment, such as inhibitors against poly ADP-ribose polymerase (PARP) molecules combined with bevacizumab for high-grade OC, after debulking surgery and platinum-based chemotherapy [2–5]. However, the curative effect is still unsatisfactory due to occult onset and no obvious early symptoms [4]. Therefore, it is urgent to clearly clarify the pathogenesis of OC and find more sensitive prognostic indicators, as well as more effective treatment methods.

DNA methylation, one of the most prevalent types of epigenetic modifications, has been shown to play important roles in a variety of physiological and pathological processes, including benign and malignant ovarian diseases [6–8]. Recently, it has been reported that DNA methylation status, as well as DNA methylation-related genes, can be used in the diagnosis, treatment and prognosis of diseases [9–11]. Meanwhile, DNA methylation probes or methylation-related genes have also been reported for the prognostic assessment of OC [12, 13]. However, the predictive efficacy of these methylation related models is not particularly accurate, and it is not clear whether there are other values in addition to the prognostic value.

Immunotherapy is a promising therapeutic method for the treatment of a variety of tumors [14]. For example, immune checkpoint inhibitors have shown good efficacy in the treatment of lymphoma and non-small-cell lung cancer (NSCLC) [15, 16], and immune checkpoint inhibitors combined with angiogenesis inhibitors and other molecular targeted drugs have also shown good efficacy in liver cancer and melanoma [17, 18]. Meanwhile, chimeric antigen receptor-T (CAR-T) cell immunotherapy has also shown impressive curative effects for hematologic malignancies [19]. Nevertheless, the efficacy of immunotherapy for OC remains limited [20, 21].

Recently, it has been reported that the DNA methylation state can affect the function of immune cells and their response to immunotherapy in some tumors [22–25]. Furthermore, it has also been reported that the efficacy of immunotherapy can be enhanced by intervening in the level of DNA methylation [26, 27]. However, the relationship between DNA methylation and immune cells in OC remains largely unknown.

In this study, a risk score signature was constructed based on 12 genes that were significantly associated with the prognosis of OC patients by systematically analyzing DNA methylation data and transcriptome data. Next, a nomogram combining the risk score and clinicopathological features was constructed for prognosis prediction. Subsequently, we determined the difference in the immune characteristics between the high- and low-risk groups through comprehensive immune analysis, which will guide the further exploration of synergistic targets to improve the efficacy of immunotherapy in OC patients.

## Material and methods

### Data acquisition and processing of the training cohort

TCGA ovarian serous cystadenocarcinoma gene expression data was measured by AffyU133a array (GPL96, *n* = 593), as well as the corresponding DNA methylation data (Infinium HumanMethylation27 platform, *n* = 616), were both downloaded from UCSC Xena (https://xena.ucsc.edu/) as the training cohort. After intersection analysis, 575 samples (including 567 primary tumor samples and 8 normal (non-tumor) ovarian samples) with both DNA methylation data and expression data were selected. For DNA methylation data, 21,676 probes without “NA” results across all the enrolled samples remained. Next, differentially methylated probes between tumor and non-tumor samples were identified by the “limma” package in R software [28]. Subsequently, Pearson correlation analysis between DNA methylation data and expression data of OC was conducted, and genes with a negative correlation between expression levels and methylation levels were identified as DNA methylation-related genes. All methods were carried out in accordance with the Declaration of Helsinki guidelines.

### Functional enrichment analysis

Gene Ontology (GO) and Kyoto Encyclopedia of Genes and Genomes (KEGG) pathway enrichment analyses of the DNA methylation-related genes were conducted by the “clusterProfiler” package [29].

### Acquisition and processing of the clinicopathological features

The clinical data of the OC patients from the training cohort were downloaded from the cBioPortal (https://www.cbioportal.org/). A total of 484 patients with complete clinicopathological data (including age, stage, histological grade, longest dimension, tumor site, race and survival data) and overall survival (OS) time greater than one month were selected for further analysis. All of the clinicopathological features were divided into two categories: age (≥ 60 y, < 60 y), stage (stage I-II, stage III-IV), histological grade (G1-G2, G3-G4), longest dimension (≥ 1 cm, < 1 cm), tumor site (unilateral, bilateral) and race (white, non-white). The clinicopathological features of TCGA-OC patients are summarized in Table S1.

### Screening of the prognostic DNA methylation-related genes and the construction of risk score signature

Prognostic values of these DNA methylation-related genes were evaluated by least absolute shrinkage and selection operator (LASSO) method using the “glmnet” package with “nfold = 10” [30], “lambda.min” was used to screen prognostic genes. Next, the prognostic genes were further screened by univariate and multivariate Cox methods (Table S2), and then the risk score signature was constructed according to the most prognostic genes (*P* < 0.05) and their corresponding risk coefficients calculated by Cox analyses (Table S3). Subsequently, the prognostic value of the signature was evaluated by Kaplan–Meier plotter curve and receiver operating characteristic curve (ROC) analyses, which were performed by the “survival”, “survminer” and “timeROC” packages [31].

### External validation of the prognostic value of the risk score signature

Microarray data and the corresponding survival data of OC patients from the GSE9891 (*n* = 276) and GSE32062 (*n* = 260) datasets were downloaded from the GEO database (https://www.ncbi.nlm.nih.gov/geo/) as validation cohorts [32, 33]. The prognostic values of the risk score signature was further validated by these two GEO datasets by Kaplan–Meier plotter analysis.

### Evaluation of the prognostic values of the clinicopathological features

To further investigate the prognostic values of the clinicopathological features in OC patients, univariate and multivariate Cox regression analyses were performed. As a result, significant prognostic clinicopathological features combined with the risk score were used for the construction of the nomogram by the “rms” package in R software. Clinical subgroup analysis was performed by the “forestplot” package in R software.

### Gene set enrichment analysis (GSEA) and immune analysis

GSEA was performed between the high- and low-risk groups of OC patients in the training cohort using the “clusterProfiler” package. Immune cell component analysis was conducted by CIBERSORT, while immune scores and tumor purity were calculated by the “estimate” package [34]. Potential drugs targeting the prognostic genes were screened preliminarily by CellMiner, a web tool based on the NCI-60 cell line set(38). Subsequently, functional modules in the two groups were identified by weighted gene co-expression network analysis (WGCNA) through integrated analysis of transcriptome data and immune cell abundances data [35]. Next, interactions between immune cells and functional modules were evaluated by correlation analysis. Before integrated analysis, immune cells expressed as “0” were removed, and the other 15 immune cells (memory B cells, plasma cells, memory resting CD4 T cells, follicular helper T cells, regulatory T cells, gamma delta T cells, activated NK cells, monocytes, M0 macrophages, M1 macrophages, M2 macrophages, resting dendritic cells, activated dendritic cells, activated mast cells and neutrophils) were left for further WGCNA.

### Quantitative real-time PCR (qPCR)

Six pairs of OC tumor tissues and corresponding peri-tumor tissues were collected from the Department of Obstetrics and Gynecology, The Second Xiangya Hospital of Central South University. All experiments performed in this study involving human samples were approved by the Clinical Research Ethics Committee of the Second Xiangya Hospital, Central South University. All patients have signed informed consent in accordance with the Declaration of Helsinki guidelines. Total RNA was extracted from OC tumor tissues and corresponding peri-tumor tissues using RNAiso Plus Reagent (TaKaRa, Kyoto, Japan) according to the manufacturer's instruction. The detailed procedures of qPCR were conducted as previously described [36]. Specific primers used in this study were synthesized by Tsingke (Beijing, China). GAPDH were used as internal controls. Relative expression levels of each gene were calculated according to the 2^−△Ct^ method. The primer sequences are listed in Table S4.

### Immunohistochemistry (IHC) staining

Ovarian cancer tissues and corresponding peri-tumor tissues were fixed in 10% formalin and embedded in paraffin blocks, then cut into sections. The detailed procedures of IHC were conducted as previously described [36]. At the end of the first day, tissue sections were incubated with primary antibody against CA2 (1:100, Proteintech, 16,961–1-AP) at 4 ℃ overnight.

### Statistical analysis

Statistical analysis was performed by R (version 4.1.0). Log-rank test was used for survival analyses. The continuous variables between the two groups were compared by t-test. Subgroup analysis was displayed as forest plots. Hazard ratios (HRs), 95% confidence intervals (CIs) and P-values were calculated by univariate Cox proportional hazards models of each subgroup. *P* < 0.05 was considered statistically significant.

## Results

### Identification of DNA methylation-related genes

**Fig. 1:**
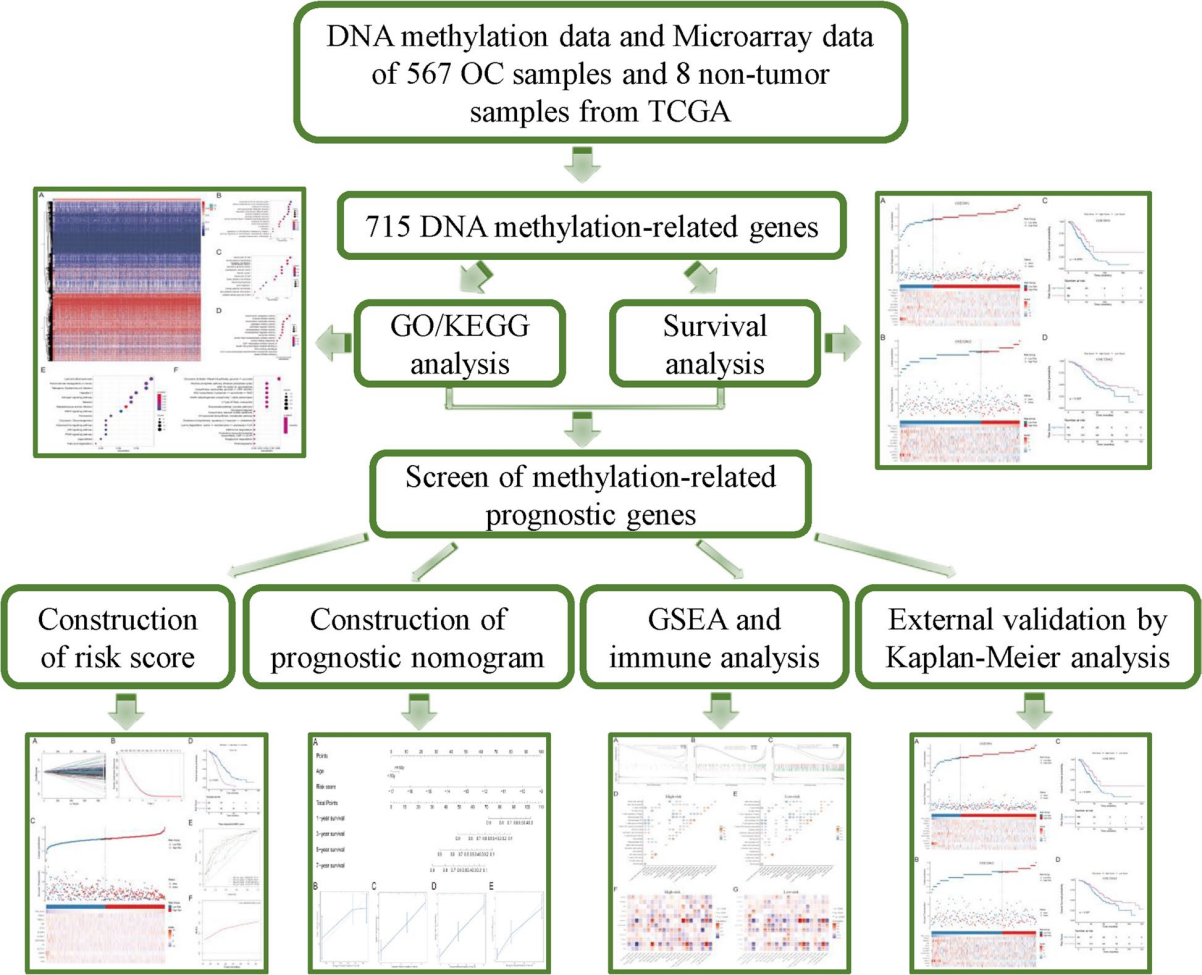
Analysis flowchart of this study

**Fig. 2:**
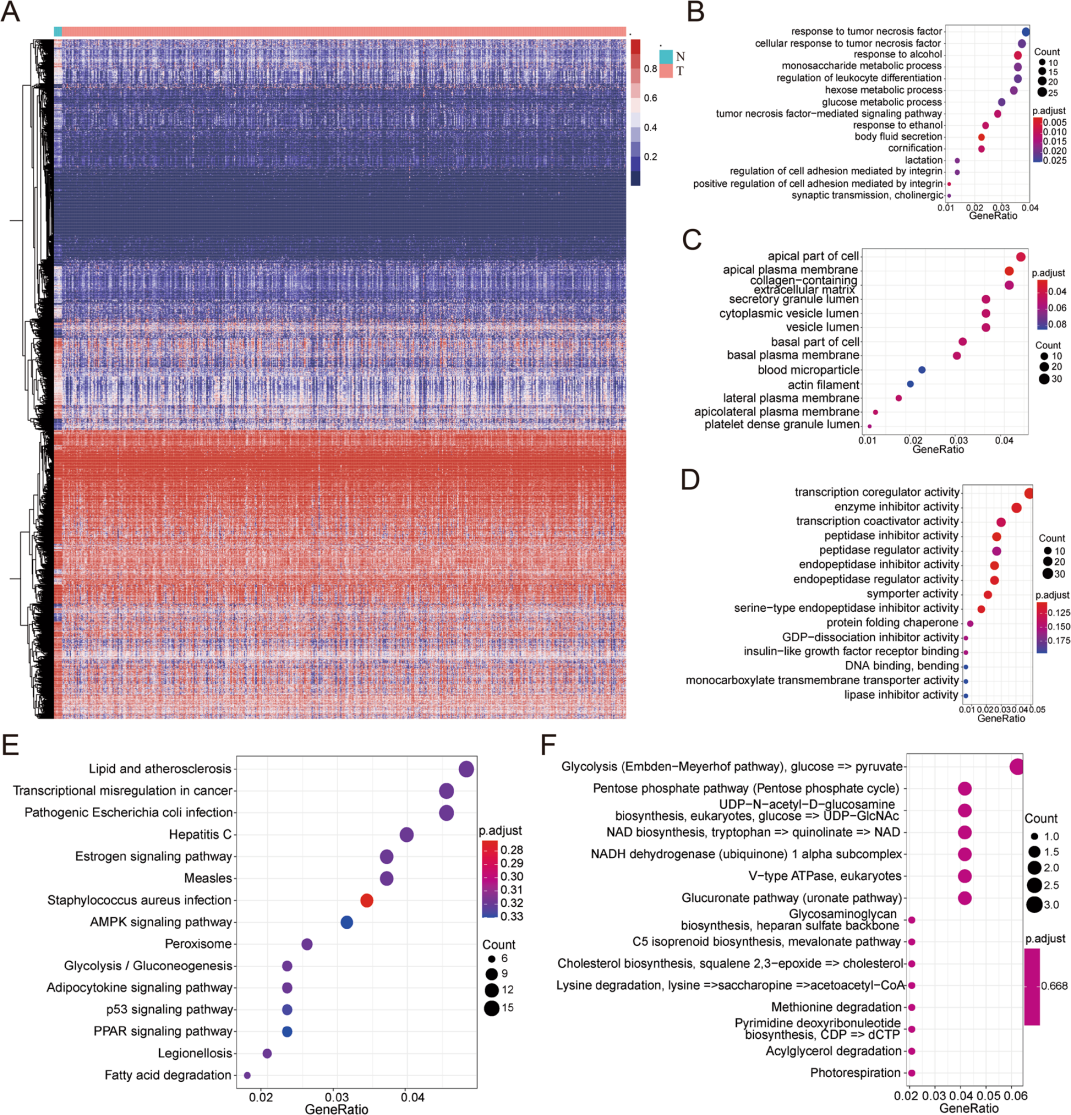
Identification and functional enrichment analysis of DNA methylation-related genes in OC. **A** Heatmap of differential methylation probes between tumor and non-tumor samples. The X axis represents samples (*N* = 8, T = 567), the Y axis represents methylation probes, the color legend represents methylation levels (β value). N: non-tumor, T: tumor. **B** Dot plot of biological process (BP) analysis. **C** Dot plot of cellular component (CC) analysis. **D** Dot plot of Molecular function (MF) analysis. **E** Dot plot of KEGG pathway analysis. **F** Dot plot of KEGG module analysis

The analysis flowchart of this study is shown in Fig. 1. By comparing 567 tumor samples with 8 non-tumor samples, 3190 differentially methylated probes (*P* < 0.05) were identified (Fig. 2A). Next, correlation analysis was conducted between DNA methylation data and expression profile data, and significant gene-probe pairs (correlation coefficient < 0 and *P* < 0.05) were selected to intersect with the previously screened differential methylation probes. Finally, 715 genes negatively correlated with methylation level were identified as methylation-related genes.

### Functional enrichment analysis

To further clarify the functional roles of the 715 methylation-related genes, enrichment analysis was conducted. The biological process (BP) results were mainly enriched in response to tumor necrosis factor, monosaccharide metabolic process, regulation of leukocyte differentiation, tumor necrosis factor-mediated signaling pathway, etc. (Fig. 2B). The cellular component (CC) results were mainly enriched in the apical part of the cell, apical plasma membrane, secretory granule lumen, cytoplasmic vesicle lumen, etc. (Fig. 2C). Molecular function (MF) was mainly enriched in transcription coregulator activity, enzyme inhibitor activity, peptidase regulator activity, symporter activity, etc. (Fig. 2D). For KEGG analysis, the results were enriched in transcriptional mis-regulation in cancer, AMPK signaling pathway, glycolysis, gluconeogenesis, p53 signaling pathway, etc. (Fig. 2E). The KEGG module analysis results showed enrichment in glycolysis, the pentose phosphate pathway, NAD biosynthesis, V-type ATPase, the glucuronate pathway, etc. (Fig. 2F).

### Construction and evaluation of the prognostic risk score signature

**Fig. 3:**
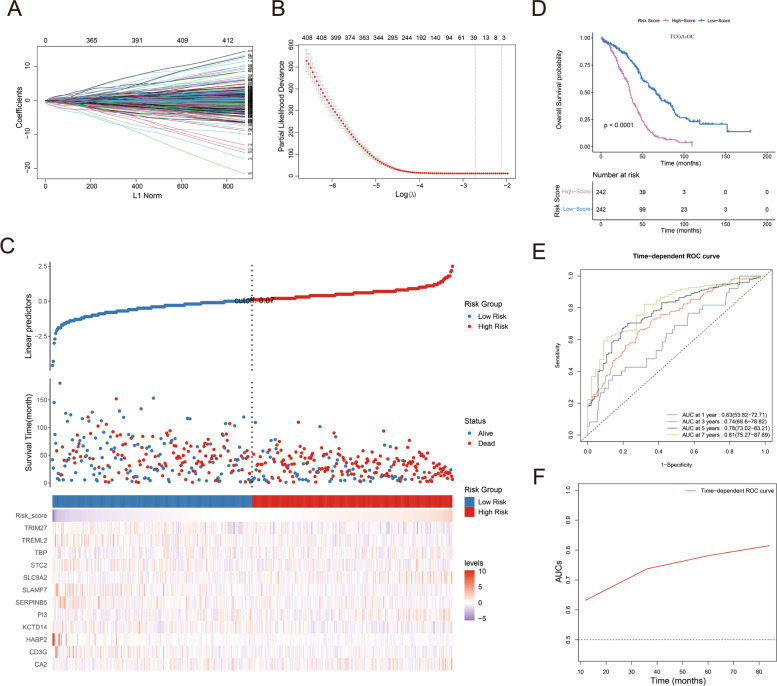
Construction and efficiency validation of the risk score. **A** Fit plot of LASSO screen. **B** Cvfit plot of LASSO screen. **C** Survival status and expression levels of the 12 prognostic genes between the two risk groups. **D** Overall survival (OS) time was compared by Kaplan–Meier plotter curve between the two risk groups. **E**, **F** Time-ROC curves of 1-, 3-, 5- and 7-years. AUCs: areas under the curves

Through LASSO screening of the 715 methylation-related genes, 36 prognostic genes were selected (Fig. 3A, B). Correlation analysis between the expression level of these 36 genes and DNA methylation level is shown in Fig. S1. Subsequently, univariate and multivariate Cox regression analyses were conducted, and 12 prognostic genes were used to construct a risk score signature for prognosis prediction. The risk score was calculated as follow: expression level of CA2 × 0.1345479 + expression level of CD3G × (-0.2689675) + expression level of HABP2 × (-1.1382079) + expression level of KCTD14 × (-0.1530112) + expression level of PI3 × 0.1185228 + expression level of SERPINB5 × (-0.2308773) + expression level of SLAMF7 × (-0.3947159) + expression level of SLC9A2 × 0.3721432 + expression level of STC2 × (-0.3173046) + expression level of TBP × 0.6366898 + expression level of TREML2 × (-1.2285490) + expression level of TRIM27 × (-0.4467591). According to the median risk score, OC patients were divided into high-risk and low-risk groups. The survival status and expression levels of the 12 prognostic genes between the two groups are presented in Fig. 3C and Fig. S2A. Next, we further verified the expression status of these 12 genes between OC tissues and corresponding peri-tumor tissues of OC patients in our center through qPCR (Fig. S3A). And the expression status of the significantly overexpressed gene CA2 in the high-risk group and OC tissues was further validated by IHC at the protein level (Fig. S3B).

As revealed by the Kaplan–Meier plotter curve, the high-risk group had a significantly poorer prognosis than the low-risk group in the training cohort (Fig. 3D). The independent prognostic values of these 12 genes were further investigated and visualized in Fig. S2B-M. Subsequently, the corresponding areas under the curves (AUCs) of the 1-, 3-, 5- and 7-year time-ROC curves were listed as 0.63, 0.74, 0.78 and 0.81, respectively (Fig. 3E), which indicates a good prognostic efficiency of this risk score signature. In addition, the red slope indicates that the prediction accuracy of this model is gradually increasing from 1-year to 7-years (Fig. 3F).

**Fig. 4:**
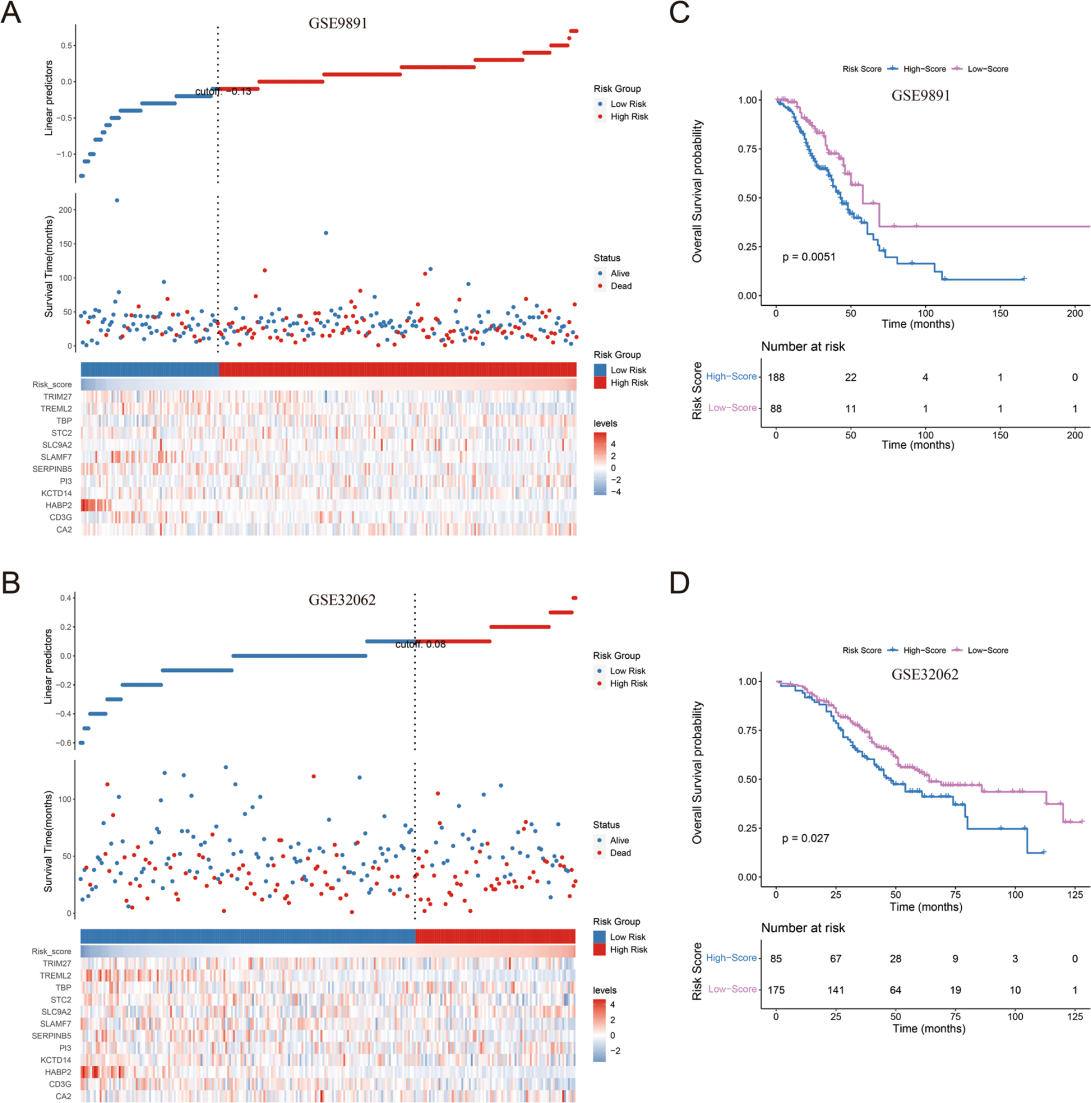
External validation of the value of the risk score for prognosis prediction. **A**, **B** Survival status and expression levels of the 12 prognostic genes between the two risk groups in the two validation cohorts (GSE9891 and GSE32062). **C** Overall survival (OS) time was compared by Kaplan–Meier plotter curve between the two risk groups in GSE9891. **D** Overall survival (OS) time was compared by Kaplan–Meier plotter curve between the two risk groups in GSE32062

Next, the prognostic efficiency of this risk score signature was further validated in two external validation cohorts (GSE9891 and GSE32062). According to the best cutoff value automatically calculated by the ROC method, patients in these two GEO datasets were also divided into high-risk and low-risk groups (Fig. 4A, B). As revealed by Kaplan–Meier plotter curves, the high-risk group also had a significantly poorer prognosis than the low-risk group in these two validation cohorts (Fig. 4C, D), consistent with the result of the training cohort.

### Evaluation of the prognostic values of the clinicopathological features

**Fig. 5:**
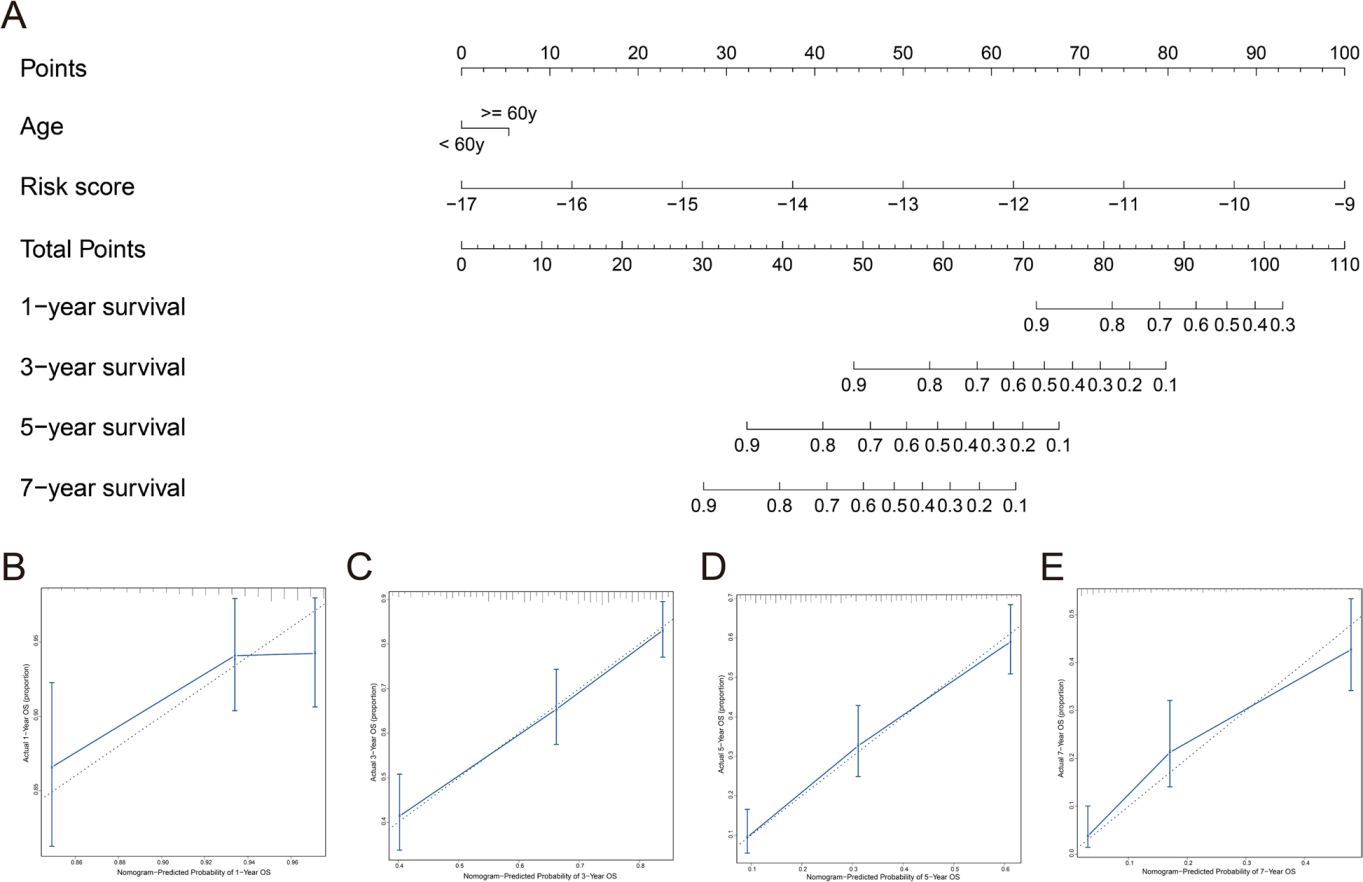
Construction and evaluation of the nomogram. **A** Nomogram to predict the 1-, 3- and 5-year survival probability of OC. **B**-**E** The results of calibration curves for 1-, 3-, 5- and 7-year OS prediction. Blue line: fitting curve, diagonal dotted line: reference line, blue error bars: 95% confidence interval (CI)

**Fig. 6:**
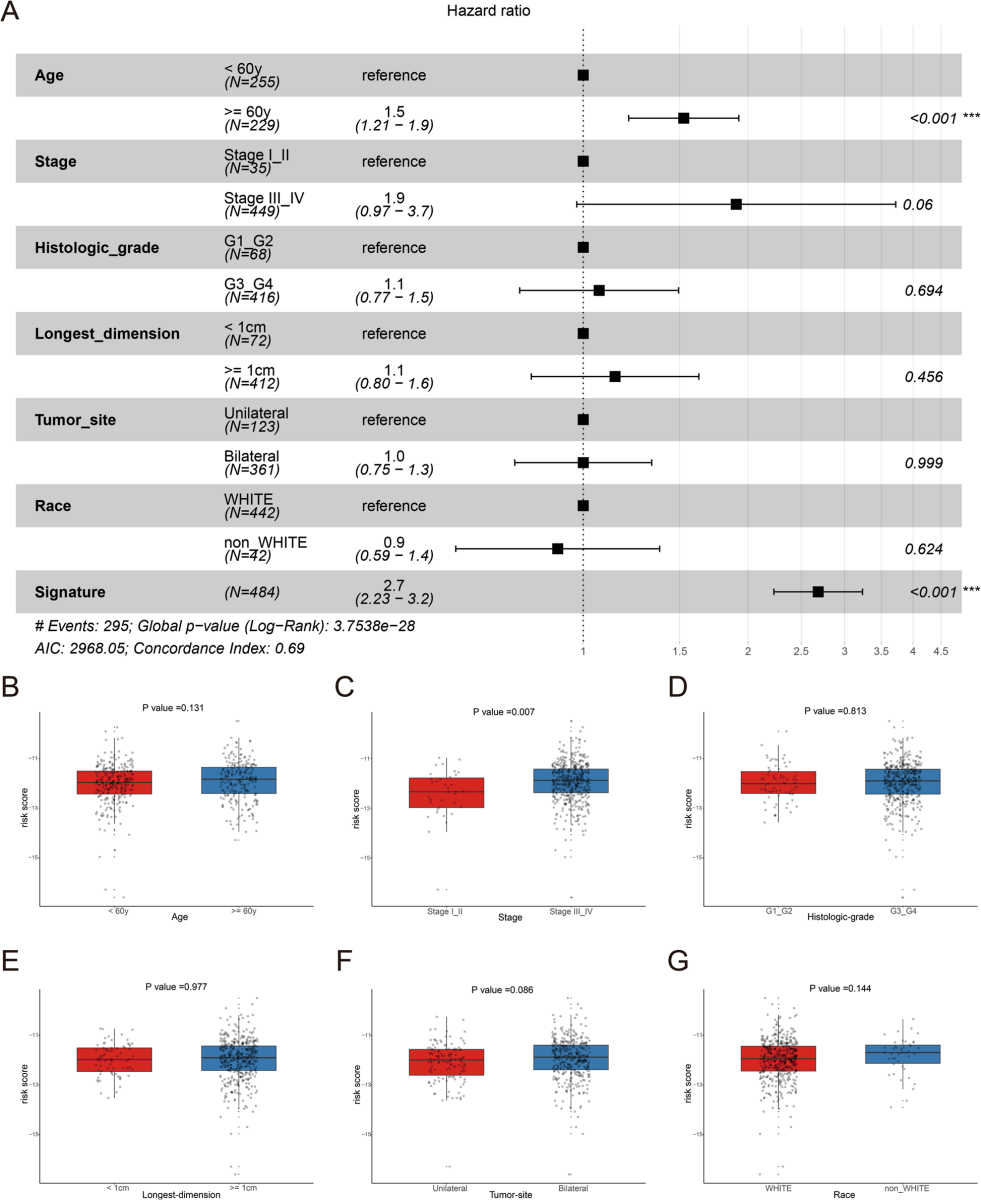
Clinical subgroup analysis. **A** Forest plot of clinicopathological features. Whisker bars indicate hazard ratio and 95%CI compared with “reference” (control) group. Boxplot of the expression levels of the risk score among different clinical subgroups including (**B**) age, (**C**) stage, (**D**) histological grade, (**E**) longest dimension, (**F**) tumor site and (**G**) race. Boxes indicate lower and upper quartile, error bars indicate maximum and minimum values, while the points outside this range indicate outliers

To investigate the prognostic values of the clinicopathological features in OC patients, univariate Cox regression analysis was performed. Features with *P* < 0.05 (age and stage) were further analyzed by multivariate regression (Table S5). As a result, age (*P* < 0.05) combined with the risk score was used for the construction of the nomogram (Fig. 5A). The results of calibration curves confirmed its good efficiency for 1-, 3-, 5- and 7-year OS prediction (Fig. 5B-E). To further elucidate the differences in the prognostic impact of different clinical subgroups, subgroup analysis was performed. As revealed in Fig. 6A, older patients (≥ 60 y) had a significantly poorer prognosis than younger patients (< 60 y). The results of the expression levels of risk scores between different subgroups showed that patients in the high-level stage had significantly higher risk scores than those in the lower stage, while there were no significant differences among other clinical subgroups (Fig. 6B-G).

### GSEA and immune analyses

**Fig. 7:**
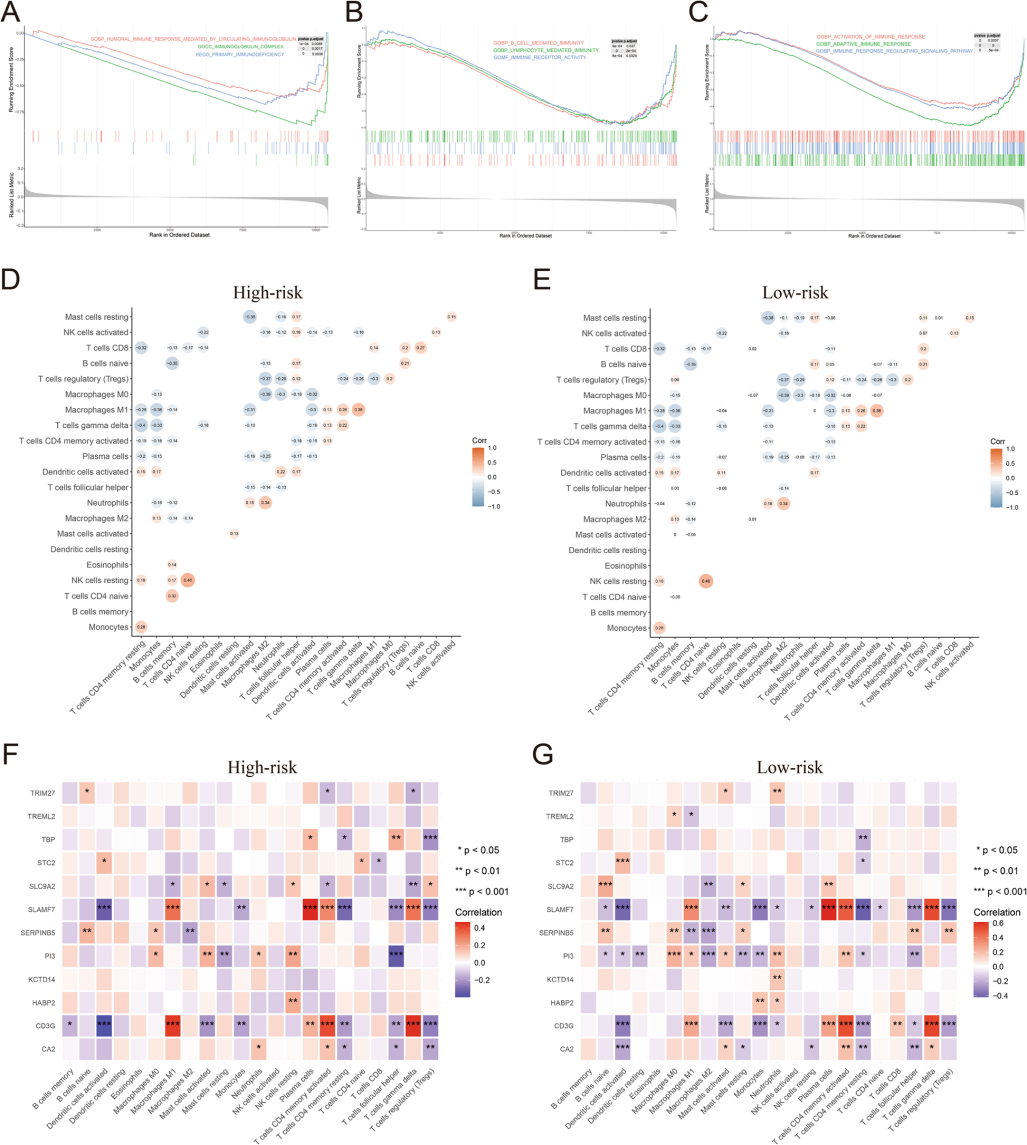
GSEA, gene and immune cell correlation analysis. **A**-**C** Immune related GSEA results between the high- and low-risk groups. X axis represent ranks of genes in ordered dataset, and Y axis represents running enrichment score of genes. **D** Correlation between immune cells in the high-risk group. **E** Correlation between immune cells in the low-risk group. **F** Correlation between immune cells and risk score genes in the high-risk group. **G** Correlation between immune cells and risk score genes in the low-risk group

To elucidate the functional differences between the high- and low-risk groups, GSEA was performed. According to the results, we found that there were significant differences in immune characteristics between the two groups, such as the immune response mediated by circulating immunoglobulin, primary immunodeficiency, immune receptor activity, adaptive immune response and immune response regulating signaling pathway (Fig. 7A-C). To further systematically explore the difference in the immune landscape between the two groups, immune cell components and immune scores were analyzed. The results showed that several immune cells were significantly differentially expressed between the two groups, including activated dendritic cells, neutrophils, plasma cells and resting memory CD4 T cells (Fig. S4A, B), while there were no significant differences in the ESTIMATE score, immune score, stomal score or tumor purity (Fig. S4C-F). Considering that the expression difference of most immune cells between the high- and low-risk groups is not significant, we speculate that the difference of immune characteristics revealed by GSEA results between the two groups may be realized by affecting the integrated function of immune cells.

To address this hypothesis, correlation analysis was conducted. The results showed that the relationships between immune cells in the two groups were similar, some strong cell correlations existed in both the high- and low-risk groups, such as resting NK cells positively correlated with naive CD4 T cells and M0 macrophages negatively correlated with M2 macrophages (Fig. 7D, E). Furthermore, correlations between immune cells and risk score genes were analyzed in the two groups. The results suggested that SLAMF7 and CD3G were closely related to a series of immune cells (such as plasma cell, memory CD4 T cells, follicular helper T cells, regulatory T cells and gamma delta T cells) in both the high- and low-risk groups (Fig. 7F, G). However, there were also significant differences between the two groups in the correlation between some genes and immune cells, such as TBP with regulatory T cells in the high-risk group, as well as CA2 with activated dendritic cells and SLC9A2 with naive B cells in the low-risk group (Fig. 7F, G). These prognostic genes closely related with immune cells may be potential synergistic targets to affecting the function of immune cells, so as to improve the efficacy of immunotherapy in OC patients. To screen potential synergistic drugs to improve the efficacy of immunotherapy, potential drugs targeting these hub genes were investigated by CellMiner (Table S6).

### Correlation between functional modules and immune cells

**Fig. 8:**
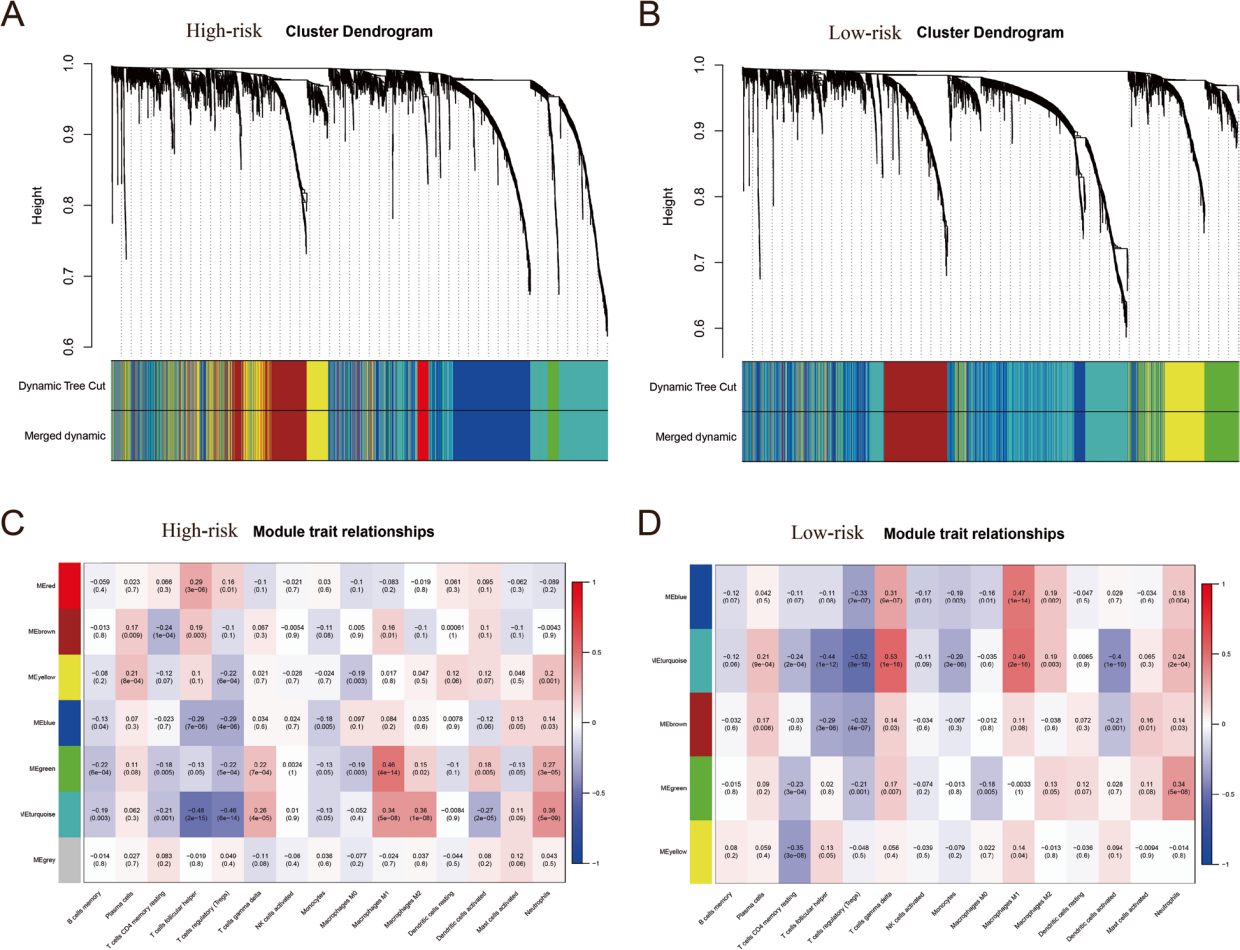
WGCNA and module analysis. **A** The result of WGCNA in the high-risk group. **B** The result of WGCNA in the low-risk group. **C** Correlation between immune cells and functional modules in the high-risk group. **D** Correlation between immune cells and functional modules in the low-risk group

To further clarify the differences between the high- and low-risk groups, WGCNA was conducted. According to the results, we found that there were great differences in the distribution of expression modules between the two groups (Fig. 8A, B). Next, correlation analysis was conducted between the functional modules and immune cells in the two groups. We found that correlations between immune cells and functional modules were also different between the two groups. For example, M1 macrophages were significantly positively correlated with green modules in the high-risk group, while gamma delta T cells and M1 macrophages were positively associated with turquoise modules in the low-risk group (Fig. 8C, D). In addition, some significant correlations also existed in the two groups, such as follicular helper T cells and regulatory T cells were significantly negatively associated with turquoise modules (Fig. 8C, D). In this part, different functional modules are composed of different gene lists. The results of WGCNA further confirmed that the difference of immune characteristics between the two risk groups may be achieved by the integrated roles between genes and immune cells.

## Discussion

In recent years, the role of epigenetic modifications in the pathogenesis of diseases has been gradually clarified [37, 38]. As an important type of epigenetic modification, DNA methylation has been reported to participate in the process of disease occurrence, diagnosis, prognosis evaluation and treatment [10, 22, 39, 40]. In OC, DNA methylation also shows value in prognostic evaluation, but the prediction efficiency is not particularly accurate, and whether there are other values remains unclear [12, 13].

In this study, DNA methylation-related genes were identified by comprehensively analyzing the DNA methylation data and transcriptome data of OC patients. Next, a risk score signature for prognosis prediction was constructed based on the 12 prognostic genes (CA2, CD3G, HABP2, KCTD14, PI3, SERPINB5, SLAMF7, SLC9A2, STC2, TBP, TREML2 and TRIM27) screened by LASSO, as well as subsequent univariate and multivariate Cox analyses. After that, the prognostic value of this risk score signature was further validated in large samples by survival analysis in the training cohort (*n* = 484) and two validation cohorts, GSE9891 (*n* = 276) and GSE32062 (*n* = 260). Furthermore, to comprehensively analyze the impact of the risk score and clinicopathological features on prognosis, a prognostic nomogram was constructed.

Among the 12 prognostic genes, there is no relevant research on CA2 (carbonic anhydrase II), CD3G, HABP2 (hyaluronan binding protein 2), KCTD14 (potassium channel tetramerization domain containing 14), PI3 (peptidase inhibitor 3), SERPINB5 (serpin peptidase inhibitor, ovalbumin member 5) and TREML2 (triggering receptor expressed on myeloid cells-like 2) in OC.

Except for these genes, SLAMF7 has been identified as a prognostic gene in OC combined with other noncoding RNAs and genes [41]. SLC9A2 (solute carrier family 9, member 2 of subfamily A) has been reported to be drug resistant in OC cell lines [42]. STC2 (stanniocalcin 2) has been reported to participate in tumor proliferation in vitro and in vivo [43]. TBP (TATA box binding protein) has been reported to participate in the transcriptional regulation of OC [44]. TRIM27 (tripartite motif containing 27) has also been reported to exert roles in the cell proliferation and chemosensitivity of OC [45, 46]. However, the value of combined analysis of these genes in OC remains largely unknown.

Immunotherapy is a promising therapeutic method for a variety of tumors [14–16], but the response of OC patients to immunotherapy remains very limited [20, 21]. Considering that it has been reported that the DNA methylation state can affect the function of immune cells and their response to immunotherapy [22–25], we systematically analyzed the relationship between the risk score signature constructed by the methylation-related genes and immune landscape. The GSEA results showed that there were significant differences in immune-related gene sets between the high- and low-risk score groups. Furthermore, the results of immune cell component analysis revealed that the proportions of some immune cells between the two groups were significantly different. According to the results of the correlation analysis, the correlation differences between immune cells and prognostic genes and gene function modules were systematically identified between the two groups. Taken together, the differences of immune characteristics between the high- and low-risk groups had been confirmed by GSEA in this study. Reasons for these differences were preliminarily elucidated by the results of immune cell components analysis and correlation analyses. These prognostic genes may be potential synergistic targets to affecting the function of immune cells, so as to improve the efficacy of immunotherapy in OC patients.

There are still some limitations in our study. First, the risk score signature was constructed based on 12 genes quantified by microarray, which increases the difficulty of its clinical application. Second, how to improve the efficacy of immunotherapy according to the relationships between DNA methylation-related genes and immune cells needs to be verified by further experimental research and clinical trials.

## Conclusion

In summary, a novel efficient DNA methylation-related gene risk score signature and a prognostic nomogram were constructed in our study for the 1-, 3-, 5- and 7-year OS rate prediction of OC patients. In addition, the difference of immune characteristics between the high- and low-risk groups separated by the methylation-related risk score was reported for the first time in OC, which broadened our horizon of finding synergistic therapeutic targets to improve the efficacy of immunotherapy in the future.

## Supplementary Information

**Additional file 1:**  **Fig. S1.** The relationship between the expression levels of the 36 DNA methylation-related genes and DNA methylation levels in OC patients. **Fig. S2.** Differential expression status and survival analyses of the 12 DNA methylation-related genes. **Fig. S3.** Validation of the differential expression status of these 12 genes in OC tumor tissues and adjacent normal tissues of our center. **Fig. S4.** Immune analysis between the high- and low-risk groups.**Table S1.** Clinicopathological features of the TCGA-OC patients. **Table S2.** Univariate and multivariate Cox analyses of the 36 DNA methylation-related genes. **Table S3.** List of the 12 prognostic genes and their corresponding risk coefficients. **Table S4.** The primer sequences used in this study. **Table S5.** Univariate and multivariate Cox analyses of the risk score and clinicopathological features. **Table S6.** Pearson correlation between drug IC50 and gene expression.

## Data Availability

The datasets analyzed in this study are available in the TCGA and GEO databases.

## Abbreviations

OC: Ovarian cancer
PARP: Poly ADP-ribose polymerase
NSCLC: Non-small-cell lung cancer
CAR-T: Chimeric antigen receptor-T
TCGA: The Cancer Genome Atlas
GEO: Gene Expression Omnibus
GO: Gene Ontology
KEGG: Kyoto Encyclopedia of Genes and Genomes
OS: Overall survival
LASSO: Least absolute shrinkage and selection operator
ROC: Receiver operating characteristic curve
GSEA: Gene set enrichment analysis
WGCNA: Weighted gene co-expression network analysis
HRS: Hazard ratios
CIs: Confidence intervals
BP: Biological process
CC: Cellular component
MF: Molecular function
