# Supplementary material for: A novel DNA methylation-related gene signature for the prediction of overall survival and immune characteristics of ovarian cancer patients
Source: J Ovarian Res. 2023 Mar 29;16:62. doi: 10.1186/s13048-023-01142-0 (PMC10053775; doi:10.1186/s13048-023-01142-0)
Supplement: Supplementary file 1 — Additional file 1: Fig. S1. The relationship between the expression levels of the 36 DNA methylation-related genes and DNA methylation levels in OC patients. Fig. S2. Differential expression status and survival analyses of the 12 DNA methylation-related genes. Fig. S3. Validation of the differential expression status of these 12 genes in OC tumor tissues and adjacent normal tissues of our center. Fig. S4. Immune analysis between the high- and low-risk groups.Table S1. Clinicopathological features of the TCGA-OC patients. Table S2. Univariate and multivariate Cox analyses of the 36 DNA methylation-related genes. Table S3. List of the 12 prognostic genes and their corresponding risk coefficients. Table S4. The primer sequences used in this study. Table S5. Univariate and multivariate Cox analyses of the risk score and clinicopathological features. Table S6. Pearson correlation between drug IC50 and gene expression. [file 13048_2023_1142_MOESM1_ESM.pdf]

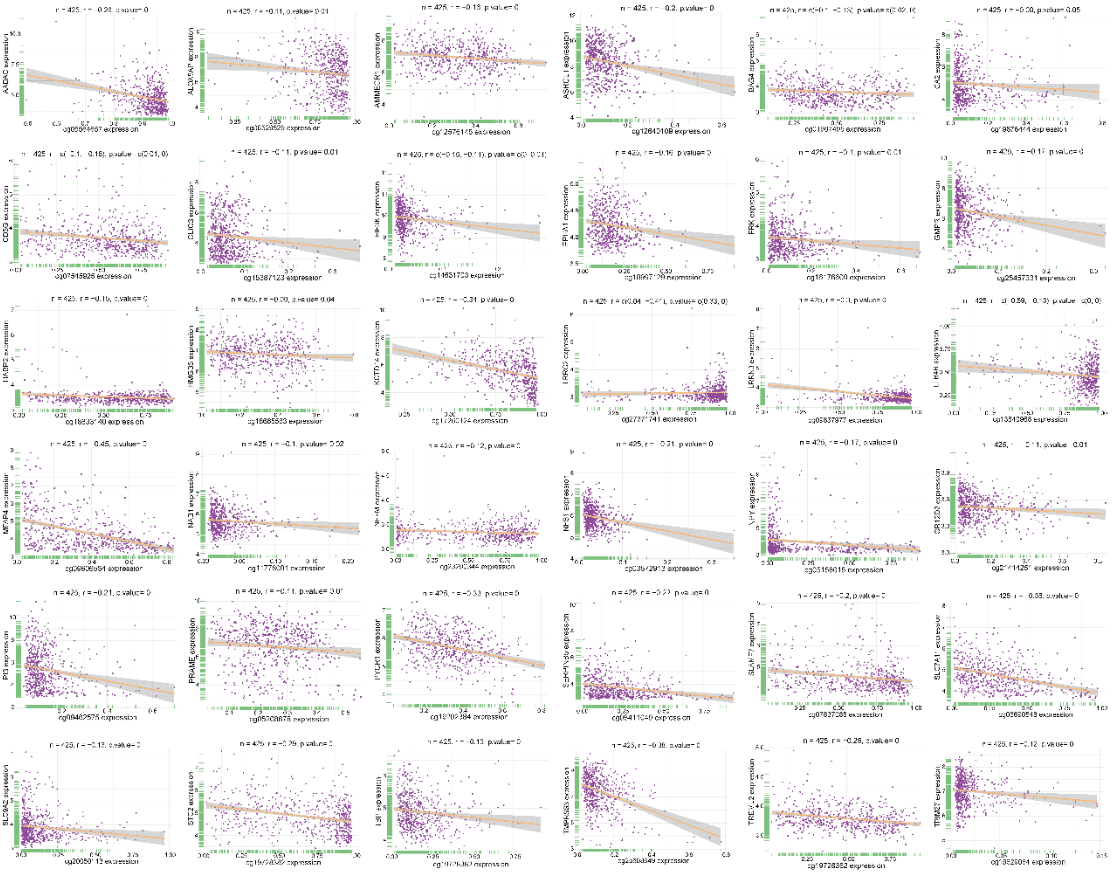

Fig.S1. The relationship between the expression levels of the 36 DNA methylation-related genes and DNA methylation levels in OC patients.

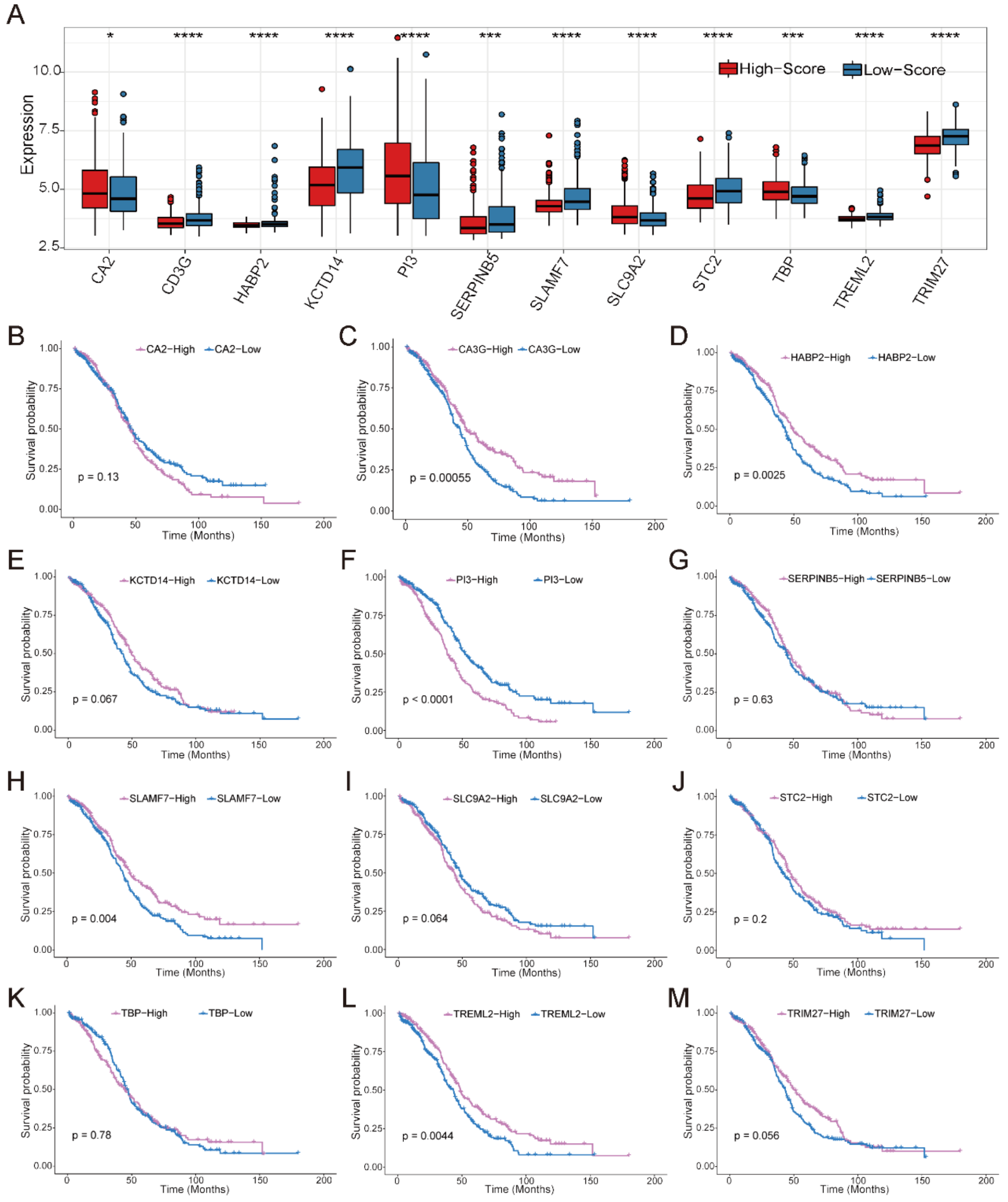

Fig.S2. Differential expression status and survival analyses of the 12 DNA methylation-related genes. (A) Boxplots visualized the differential expression status of the 12 DNA methylation-related genes between the high- and low-risk groups. (B-M) OS time was compared by Kaplan-Meier plotter curve between the high- and low-expression groups of CA2, CD3G, HABP2, KCTD14, PI3, SERPINB5, SLAMF7, SLC9A2, STC2, TBP, TREML2 and TRIM27. *p < 0.05, **p < 0.01, ***p < 0.001, ****p < 0.0001.

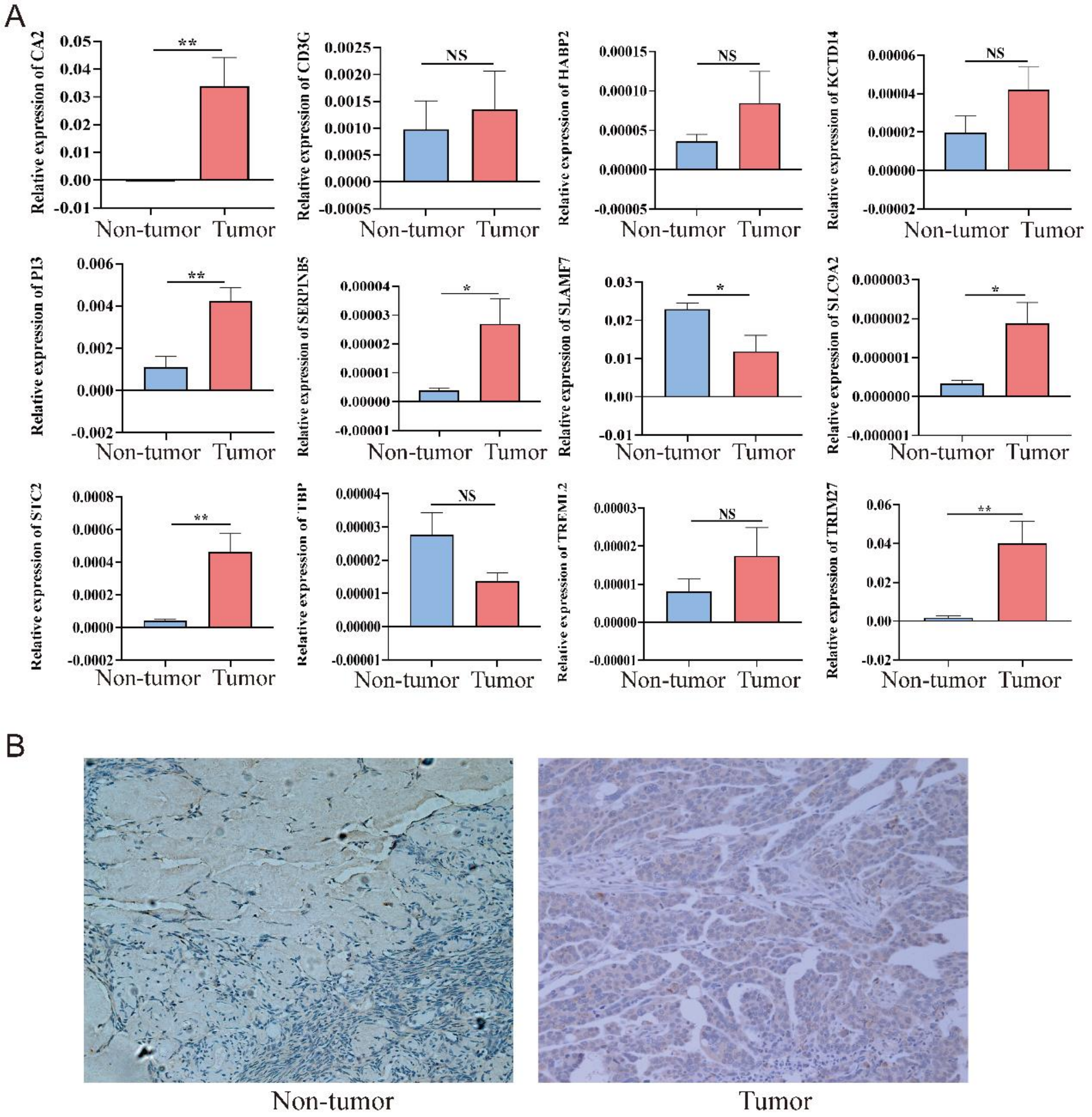

Fig.S3. Validation of the differential expression status of these 12 genes in OC tumor tissues and adjacent normal tissues of our center. (A) Boxplots visualized the differential expression status of the 12 DNA methylation-related genes between the non-tumor and tumor samples (n = 6). (B) IHC result verified the differential expression status of CA2 in OC tumor tissues and adjacent normal tissues (200X). *p < 0.05, **p < 0.01, NS: not significant.

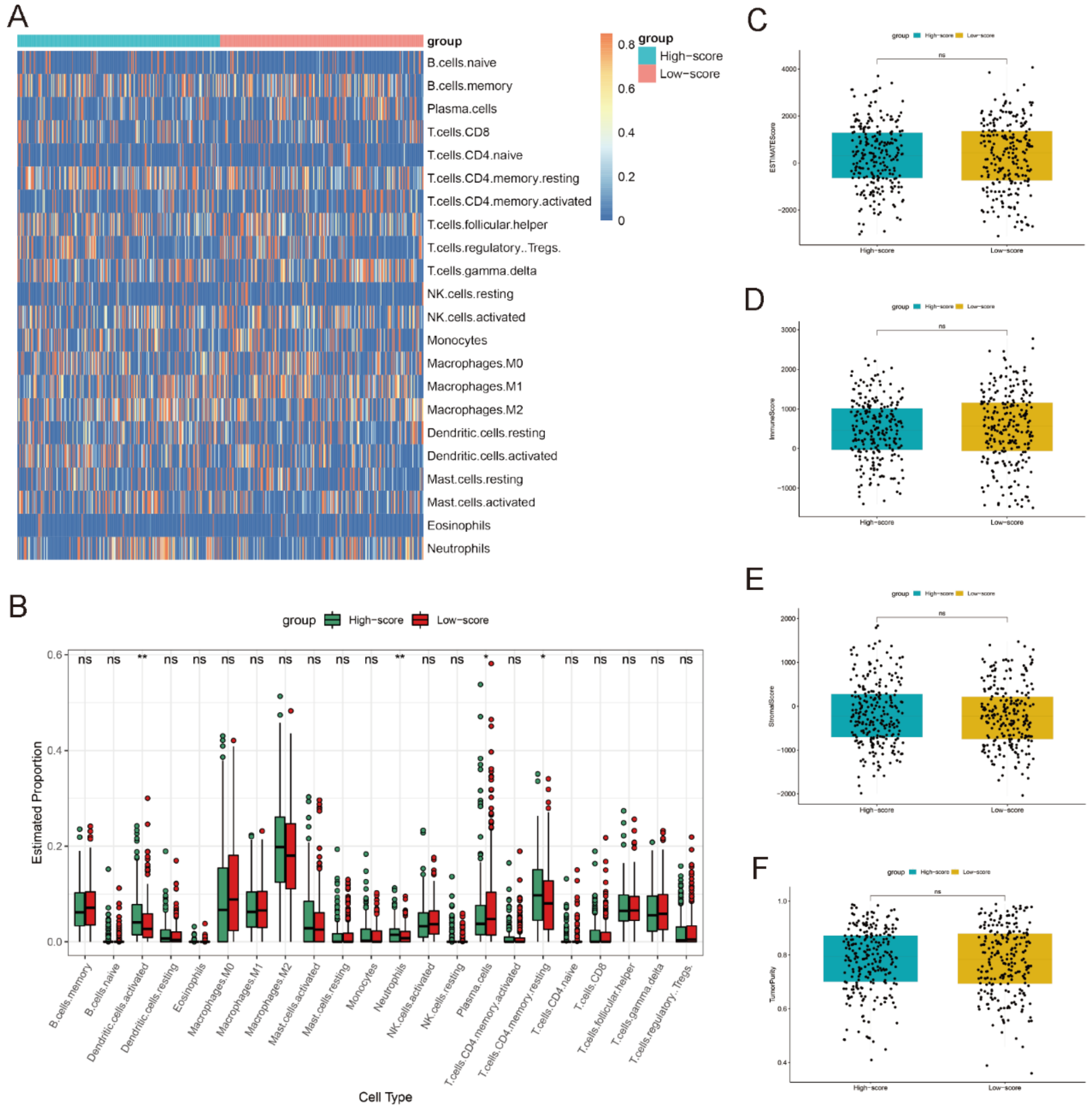

Fig.S4. Immune analysis between the high- and low-risk groups. (A) Heatmap of the differentiated immune cells. The color legend represents the abundances of immune cells. (B) Histogram of the differentiated immune cells. (C-F) Boxplot plot of estimate score, immune score, stomal score and tumor purity. Boxes indicate lower and upper quartile, error bars indicate maximum and minimum values, while the points outside this range indicate outliers. *p < 0.05, **p < 0.01, ns: not significant.

Clinicopathological features	Frequency (%)
Age	
< 60 y	255 (53%)
≥ 60 y	229 (47%)
Stage	
Stage I_II	35 (7%)
Stage III_IV	449 (93%)
Histologic-grade	
G1_G2	68 (14%)
G3_G4	416 (86%)
Longest-dimension	
< 1 cm	72 (15%)
≥ 1 cm	412 (85%)
Tumor-site	
Unilateral	123 (25%)
Bilateral	361 (75%)
Race	
WHITE	442 (91%)
Non-WHITE	42 (9%)

Table S1. Clinicopathological features of the TCGA-OC patients.

	Univariate analysis		Multivariate analysis	
Genes	HR (95% CI)	P	HR (95% CI)	P
AADAC	0.79 (0.7-0.9)	0	0.87 (0.75-1)	0.057
ALOX5AP	1.07 (0.99-1.15)	0.069	1.06 (0.96-1.18)	0.237
AMMECR1	0.82 (0.73-0.93)	0.003	0.97 (0.83-1.14)	0.744
ASRGL1	0.9 (0.83-0.97)	0.009	0.91 (0.82-1.01)	0.073
BAG4	1.22 (0.95-1.57)	0.118	1.24 (0.94-1.65)	0.129
**CA2**	1.13 (1.02-1.25)	0.015	1.16 (1.03-1.3)	**0.014***
**CD3G**	0.61 (0.46-0.82)	0.001	0.64 (0.42-0.98)	**0.038***
CLIC3	1.16 (1.06-1.27)	0.001	1.04 (0.93-1.16)	0.498
EIF3K	1.2 (1.01-1.43)	0.044	1.09 (0.9-1.32)	0.383
EPHA1	0.6 (0.41-0.86)	0.006	0.92 (0.58-1.46)	0.734
FRK	1.3 (1.05-1.61)	0.018	1.07 (0.83-1.39)	0.601
GMPR	0.85 (0.77-0.93)	0.001	1.06 (0.94-1.2)	0.366
**HABP2**	0.45 (0.26-0.77)	0.004	0.32 (0.17-0.6)	**0*****
HMGB3	0.78 (0.66-0.93)	0.005	0.91 (0.74-1.13)	0.405
**KCTD14**	0.9 (0.82-0.98)	0.02	0.9 (0.81-0.99)	**0.039***
LRRC2	0.75 (0.52-1.08)	0.124	0.72 (0.47-1.09)	0.117
LRRN3	1.41 (1.12-1.78)	0.004	1.19 (0.88-1.61)	0.249
LTB4R	0.64 (0.33-1.24)	0.186	0.58 (0.26-1.33)	0.201
MFAP4	1.17 (1.05-1.3)	0.005	1.02 (0.89-1.18)	0.741
NAB1	0.77 (0.64-0.94)	0.01	0.81 (0.64-1.02)	0.079
NEFM	2.56 (1.43-4.58)	0.002	1.43 (0.64-3.23)	0.384
NFS1	1.38 (1.12-1.7)	0.002	1.28 (0.99-1.65)	0.057
NPY	1.1 (1.01-1.19)	0.026	1.02 (0.93-1.13)	0.614
OR12D2	0.28 (0.13-0.62)	0.002	0.46 (0.21-1.04)	0.062
**PI3**	1.14 (1.07-1.21)	0	1.09 (1.02-1.17)	**0.013***
PRAME	0.89 (0.83-0.96)	0.002	0.95 (0.87-1.04)	0.287
PYCR1	0.74 (0.6-0.9)	0.003	0.95 (0.74-1.22)	0.697
**SERPINB5**	0.89 (0.78-1.01)	0.075	0.85 (0.72-0.99)	**0.038***
**SLAMF7**	0.76 (0.63-0.91)	0.004	0.73 (0.56-0.95)	**0.021***
SLC7A11	0.81 (0.69-0.96)	0.014	0.9 (0.72-1.11)	0.318
**SLC9A2**	1.36 (1.12-1.66)	0.002	1.39 (1.11-1.75)	**0.004****
**STC2**	0.87 (0.74-1.02)	0.082	0.78 (0.65-0.94)	**0.008****
**TBP**	1.24 (1.01-1.53)	0.04	1.49 (1.13-1.97)	**0.004****
TMPRSS3	0.91 (0.83-1)	0.04	0.94 (0.85-1.04)	0.235
**TREML2**	0.4 (0.23-0.7)	0.001	0.3 (0.16-0.57)	**0*****
**TRIM27**	0.71 (0.59-0.86)	0	0.67 (0.52-0.87)	**0.002****

*p < 0.05, **p < 0.01, ***p < 0.001.

Table S2. Univariate and multivariate Cox analyses of the 36 DNA methylation-related genes.

Gene	Full name	Risk coefficients
CA2	carbonic anhydrase II	0.1345479
CD3G	cluster of differentiation, 3G	-0.2689675
HABP2	hyaluronan binding protein 2	-1.1382079
KCTD14	potassium channel tetramerization domain containing 14	-0.1530112
PI3	peptidase inhibitor 3	0.1185228
SERPINB5	serpin peptidase inhibitor, ovalbumin member 5	-0.2308773
SLAMF7	signaling lymphocytic activation molecule family member 7	-0.3947159
SLC9A2	solute carrier family 9, member 2 of subfamily A	0.3721432
STC2	stanniocalcin 2	-0.3173046
TBP	TATA box binding protein	0.6366898
TREML2	triggering receptor expressed on myeloid cells-like 2	-1.228549
TRIM27	tripartite motif containing 27	-0.4467591

Table S3. List of the 12 prognostic genes and their corresponding risk coefficients.

CA2 F’	GAGCAGGTGTTGAAATTCCGTAAA
CA2 R’	GGAAGCTTTGATTTGCCTGTTCT
CD3G F’	AGATGATCGGCTTCCTAACTGAA
CD3G R’	TCCTGTCCAGCAATGAAGTAGAC
HABP2 F’	CAGAGCTTTAGGGTGGAGAAGAT
HABP2 R’	CAAGCACACAGTCTTCACGTATTT
KCTD14 F’	GCCACCACCCATTAGATGTCTAC
KCTD14 R’	GAACATCTCTGCCAGCTTTGAG
PI3 F’	AACCGCTGCTTGAAAGATACTGA
PI3 R’	GAGGAAGAATGGACAGTGTGGGA
SERPINB5 F’	ACCAAACCAGTGCAGATGATGAA
SERPINB5 R’	TGGAGAGTTTGACCTTGGCATTG
SLAMF7 F’	CAACATGCCTCACCCTCATCTAT
SLAMF7 R’	TGTGTTGAAGGTCCAGACAATAGA
SLC9A2 F’	AGTGTCAGCGAAACCTTGATCTT
SLC9A2 R’	AGGCAATGATGAACTGGTCCTTAA
STC2 F’	CACTGTTTGGTCAACGCTGG
STC2 R’	AGCGTGGGCCTTACATTTCA
TBP F’	GTGCTCACCCACCAACAATTTAG
TBP R’	CGTCGTCTTCCTGAATCCCTTTA
TREML2 F’	GAAGAGACACATGGCAAGCTACA
TREML2 R’	GCACAAGTCCTCCAGCTTCATAA
TRIM27 F’	ATCACACCTCCAGATTTGCAAGA
TRIM27 R’	CAGAGTCACGTCCACTGAGTATAA

Table S4. The primer sequences used in this study.

Clinicopathological features	Univariate analysis		Multivariate analysis	
	HR (95% CI)	P	HR (95% CI)	P
**Age**	1.56 (1.24-1.96)	0***	1.52 (1.21-1.92)	**0*****
Histologic-grade	1.15 (0.83-1.61)	0.398	NA	NA
Longest-dimension	1.12 (0.8-1.57)	0.52	NA	NA
Race	1.34 (0.88-2.05)	0.167	NA	NA
Stage	2.33 (1.2-4.53)	0.013*	1.94 (1-3.78)	0.05
Tumor-site	1.14 (0.86-1.5)	0.361	NA	NA
**Risk score**	2.72 (2.26-3.27)	0***	2.69 (2.23-3.24)	**0*****

***P < 0.001.

Table S5. Univariate and multivariate Cox analyses of the risk score and clinicopathological features.

Gene	Drug	correlation	P value
CD3G	Nelarabine	0.891799	1.23E-21
TREML2	Nelarabine	0.875825	5.29E-20
CD3G	Chelerythrine	0.648366	2.13E-08
CD3G	XK-469	0.645979	2.49E-08
TREML2	Fluphenazine	0.626415	8.63E-08
CD3G	Bendamustine	0.625175	9.31E-08
TREML2	Chelerythrine	0.612409	1.99E-07
TREML2	Cyclophosphamide	0.543996	7.04E-06
CD3G	Asparaginase	0.542698	7.48E-06
TREML2	Fenretinide	0.530136	1.32E-05
STC2	Alvespimycin	-0.51347	2.72E-05
TRIM27	Vorinostat	0.513071	2.77E-05
TREML2	Dexamethasone Decadron	0.512503	2.84E-05
CD3G	Batracylin	0.50796	3.43E-05
STC2	Dabrafenib	-0.50073	4.61E-05
TREML2	Hydroxyurea	0.496872	5.39E-05
TREML2	PX-316	0.489233	7.29E-05
TREML2	Asparaginase	0.487061	7.93E-05
SLAMF7	LMP776	-0.4852	8.52E-05
TREML2	Irofulven	-0.48437	8.80E-05
CD3G	Fenretinide	0.482693	9.39E-05
STC2	geldanamycin analog	-0.48077	0.000101
SLC9A2	Acetalax	0.479683	0.000105
STC2	Tanespimycin	-0.47909	0.000108
CD3G	Chlorambucil	0.4763	0.00012
STC2	Bafetinib	-0.46991	0.000152
TRIM27	Fludarabine	0.46829	0.000161
CD3G	Ifosfamide	0.467413	0.000166
TREML2	Imexon	0.465977	0.000175
TBP	Chelerythrine	0.463081	0.000195
TREML2	Arsenic trioxide	0.462874	0.000196
TREML2	XK-469	0.445683	0.000359
STC2	Dolastatin 10	-0.44552	0.000361
STC2	Eribulin mesilate	-0.4449	0.000369
STC2	Vemurafenib	-0.44482	0.00037
TRIM27	Cladribine	0.438369	0.00046
TREML2	Ifosfamide	0.435764	0.000501
SLAMF7	Dabrafenib	0.435235	0.00051
CD3G	Fluphenazine	0.433005	0.000549
CD3G	Pipobroman	0.432828	0.000552
CA2	Afatinib	0.432755	0.000554
TREML2	Carmustine	0.431752	0.000572
CD3G	Melphalan	0.42637	0.000682

TREML2	Dimethylaminoparthenolide	0.425387	0.000704
SLC9A2	bisacodyl, active ingredient of viraplex	0.42537	0.000704
CD3G	Uracil mustard	0.424691	0.000719
CD3G	PX-316	0.422211	0.000779
CD3G	Dexamethasone Decadron	0.420998	0.000809
SLC9A2	Epothilone B	-0.41991	0.000838
STC2	By-Product of CUDC-305	-0.41789	0.000893
SLAMF7	Selumetinib	0.413709	0.001017
CD3G	Fludarabine	0.412146	0.001068
SLAMF7	Vemurafenib	0.411009	0.001106
STC2	AT-13387	-0.4108	0.001113
STC2	Cobimetinib (isomer 1)	-0.40923	0.001168
TREML2	Oxaliplatin	0.407001	0.00125
SERPINB5	Arsenic trioxide	-0.40691	0.001254
STC2	Selumetinib	-0.40459	0.001344
STC2	Acetalax	0.404045	0.001367
STC2	bisacodyl, active ingredient of viraplex	0.403636	0.001384
TREML2	Lomustine	0.397877	0.001643
TREML2	Fludarabine	0.397652	0.001654
CD3G	Thiotepa	0.395274	0.001774
CD3G	Triethylenemelamine	0.394806	0.001798
CD3G	Cytarabine	0.394561	0.001811
SERPINB5	kahalide f	0.394223	0.001829
SLAMF7	8-Chloro-adenosine	-0.39396	0.001843
CD3G	Hydroxyurea	0.391968	0.001953
TBP	Nelarabine	0.391825	0.001961
SLC9A2	SR16157	0.390552	0.002035
TREML2	Nilotinib	0.390502	0.002038
TREML2	Chlorambucil	0.386365	0.002295
TREML2	Imatinib	0.384935	0.00239
CD3G	Etoposide	0.384407	0.002426
TBP	Hydroxyurea	0.384044	0.002451
CD3G	Idarubicin	0.383583	0.002483
HABP2	Itraconazole	0.38257	0.002555
STC2	Nilotinib	-0.38167	0.002621
TREML2	Pipobroman	0.379468	0.002788
SLC9A2	Asparaginase	-0.3782	0.002888
STC2	Vinblastine	-0.37686	0.002997
SLC9A2	Fulvestrant	0.37664	0.003016
STC2	Hypothemycin	-0.37423	0.003223
CA2	Erlotinib	0.372769	0.003355
TRIM27	kahalide f	-0.37201	0.003425
TRIM27	Nelarabine	0.371588	0.003464
TREML2	Palbociclib	0.369798	0.003637

PI3	Asparaginase	-0.36906	0.00371
STC2	Everolimus	0.367808	0.003838
CD3G	Amonafide	0.366841	0.003939
PI3	Gemcitabine	-0.36599	0.00403
SLAMF7	Pyrazoloacridine	-0.36561	0.004071
CD3G	LMP-400	0.364775	0.004163
TREML2	BN-2629	0.364495	0.004194
TREML2	Batracylin	0.361754	0.004511
SERPINB5	Ixazomib citrate	-0.36093	0.004609
PI3	Cladribine	-0.35994	0.004731
CD3G	Pyrazoloacridine	0.358341	0.004934
CD3G	Oxaliplatin	0.357623	0.005027
KCTD14	5-fluoro deoxy uridine 10mer	-0.35725	0.005077
TREML2	Cytarabine	0.356057	0.005236
TREML2	Uracil mustard	0.353131	0.005648
SLAMF7	PD-98059	0.353063	0.005657
TREML2	Melphalan	0.35271	0.005709
TREML2	LY-294002	-0.35247	0.005744
STC2	Sonidegib	0.350819	0.005992
SLAMF7	Cobimetinib (isomer 1)	0.349986	0.006121
HABP2	Vismodegib	0.347226	0.006565
SLAMF7	Hypothemycin	0.344895	0.006961
TREML2	Idarubicin	0.343878	0.007141
CD3G	Dexrazoxane	0.341716	0.007536
STC2	Homoharringtonine	-0.34156	0.007565
TREML2	Calusterone	0.340511	0.007764
CD3G	Teniposide	0.340114	0.00784
SLC9A2	Pipamperone	-0.3379	0.00828
STC2	Trametinib	-0.3376	0.00834
TREML2	Seliciclib	-0.33751	0.008358
TREML2	Dacarbazine	0.336841	0.008497
CD3G	7-Hydroxystaurosporine	0.336718	0.008522
CD3G	Carmustine	0.336584	0.00855
TREML2	Etoposide	0.336407	0.008587
SERPINB5	Cisplatin	-0.33618	0.008635
TREML2	Vorinostat	0.336021	0.008669
SERPINB5	Perifosine	0.335028	0.008881
STC2	Triciribine phosphate	0.333343	0.009251
STC2	Olaparib	0.331929	0.009572
CD3G	Lomustine	0.331515	0.009667
TRIM27	Mithramycin	-0.33137	0.009701
CD3G	Arsenic trioxide	0.330784	0.009838
TREML2	Bendamustine	0.32993	0.010042
CD3G	Valrubicin	0.329688	0.0101

TRIM27	Cyclophosphamide	0.327517	0.010636
STC2	Crizotinib	-0.32706	0.010752
CD3G	Irinotecan	0.326177	0.010979
TRIM27	Fenretinide	0.326164	0.010982
TREML2	Raltitrexed	0.324031	0.011547
TREML2	Thiotepa	0.32381	0.011607
STC2	ABT-199	-0.32297	0.011838
SLAMF7	Bafetinib	0.322863	0.011868
CD3G	Digoxin	0.322539	0.011958
TREML2	Amonafide	0.322372	0.012004
TREML2	Axitinib	0.321319	0.012303
TREML2	Dexrazoxane	0.32124	0.012325
HABP2	Imiquimod	0.320297	0.012598
TREML2	3-Bromopyruvate (acid)	0.320126	0.012648
PI3	tfdu	-0.31995	0.012699
CD3G	BN-2629	0.319669	0.012783
CD3G	Dimethylaminoparthenolide	0.318908	0.01301
STC2	Vincristine	-0.31823	0.013214
TREML2	Cladribine	0.317716	0.013372
TRIM27	Allopurinol	0.316818	0.01365
STC2	Zoledronate	0.316488	0.013754
SLC9A2	Carmustine	-0.31559	0.014041
SLAMF7	Batracylin	-0.31515	0.01418
PI3	Clofarabine	-0.31484	0.014283
SERPINB5	Gemcitabine	-0.31404	0.014544
CD3G	Cladribine	0.312086	0.015202
TRIM27	Depsipeptide	-0.31101	0.015577
CA2	Aminoflavone	0.309255	0.016201
CD3G	Irofulven	-0.30856	0.016456
CA2	Vemurafenib	-0.30806	0.016639
SLC9A2	Staurosporine	-0.30788	0.016705
CA2	Ibrutinib	0.307679	0.016781
TREML2	Triethylenemelamine	0.307222	0.016953
STC2	Pipamperone	-0.30711	0.016993
TBP	kahalide f	-0.3071	0.016999
SERPINB5	Carboplatin	-0.30676	0.017129
TREML2	Raloxifene	0.306651	0.017169
CD3G	Palbociclib	0.306594	0.017191
STC2	Staurosporine	0.305596	0.017575
CD3G	Vorinostat	0.305126	0.017759
STC2	Dasatinib	0.304784	0.017893
TRIM27	Chelerythrine	0.304671	0.017938
PI3	Etoposide	-0.30267	0.018746
CA2	Tyrothricin	-0.30238	0.018865

TBP	Cytarabine	0.302165	0.018952
KCTD14	Elliptinium Acetate	-0.30166	0.019164
SERPINB5	Uracil mustard	-0.30158	0.019197
TRIM27	Hydroxyurea	0.301496	0.019231
CA2	Actinomycin D	-0.30124	0.019341
TBP	LMP776	0.301056	0.019417
SLAMF7	Clofarabine	-0.30077	0.019537
SLAMF7	By-Product of CUDC-305	-0.30038	0.019703
SERPINB5	tfdu	-0.29993	0.019896
STC2	Rapamycin	0.299802	0.019954
STC2	Cyclophosphamide	-0.29904	0.020284
TREML2	Fostamatinib	0.298659	0.020454
CD3G	Acrichine	0.298419	0.02056
SERPINB5	Bortezomib	-0.29823	0.020643
STC2	Tegafur	-0.29795	0.02077
PI3	Vorinostat	-0.29743	0.021003
TREML2	LMP-400	0.29737	0.021031
CD3G	Nitrogen mustard	0.295745	0.021777
CD3G	Raltitrexed	0.295706	0.021796
STC2	PD-98059	-0.29515	0.022058
TBP	Vemurafenib	-0.2949	0.022175
TREML2	Pyrazoloacridine	0.294819	0.022213
PI3	Teniposide	-0.29478	0.022229
TBP	Asparaginase	0.294479	0.022375
SLC9A2	By-Product of CUDC-305	0.293505	0.022844
TREML2	7-Hydroxystaurosporine	0.29326	0.022963
SLC9A2	okadaic acid	-0.29274	0.023217
SERPINB5	Carmustine	-0.29262	0.023275
SLC9A2	Selumetinib	-0.29	0.0246
TBP	8-Chloro-adenosine	0.28962	0.024798
STC2	Pralatrexate	-0.28958	0.02482
HABP2	geldanamycin analog	-0.28945	0.024885
SLAMF7	Docetaxel	-0.28837	0.025455
KCTD14	Fluorouracil	-0.28814	0.025581
STC2	Sunitinib	-0.2868	0.026301
SLAMF7	Mitoxantrone	-0.28663	0.026395
TREML2	Daunorubicin	0.286278	0.02659
SERPINB5	Chlorambucil	-0.28565	0.026941
HABP2	Nelfinavir	0.285309	0.027129
TREML2	Teniposide	0.284157	0.027783
KCTD14	Mitomycin	-0.28404	0.027851
TBP	Cordycepin	0.284011	0.027866
CA2	Bafetinib	-0.28361	0.028095
CD3G	Epirubicin	0.283606	0.0281

TBP	Cladribine	0.283099	0.028394
SERPINB5	Melphalan	-0.28301	0.028448
PI3	Cytarabine	-0.283	0.02845
SERPINB5	Etoposide	-0.28241	0.028798
CA2	Elliptinium Acetate	-0.2824	0.028802
SLC9A2	Ixazomib citrate	-0.28167	0.02924
TBP	Fenretinide	0.281661	0.029243
SLAMF7	Dasatinib	-0.28152	0.029331
TBP	Fostamatinib	0.281185	0.029529
TRIM27	Actinomycin D	-0.28117	0.02954
TRIM27	6-Mercaptopurine	0.280735	0.029802
TREML2	Pipamperone	0.280704	0.02982
CA2	Doxorubicin	-0.28049	0.029952
SERPINB5	Ifosfamide	-0.28041	0.030001
TREML2	Valrubicin	0.280158	0.030154
CD3G	Daunorubicin	0.279707	0.030431
SLC9A2	1st Precursor Intermediate to TDP 665759	0.278974	0.030887
HABP2	Seliciclib	0.277624	0.031742
CD3G	Imexon	0.277508	0.031816
STC2	1st Precursor Intermediate to TDP 665759	0.277357	0.031914
SLC9A2	Carboplatin	-0.27735	0.031916
SLAMF7	ABT-199	0.27725	0.031983
TREML2	Simvastatin	-0.27725	0.031985
CD3G	Cyclophosphamide	0.277068	0.0321
TREML2	kahalide f	-0.2767	0.032341
CA2	Teniposide	-0.27643	0.032512
PI3	Calusterone	0.275982	0.032808
CA2	AFP464	0.275971	0.032816
TRIM27	Parthenolide	0.275485	0.033137
SERPINB5	Lapachone	-0.27533	0.033241
SLAMF7	XL-147	0.275021	0.033446
TBP	Dimethylaminoparthenolide	0.275002	0.033459
SLAMF7	Midostaurin	-0.27469	0.033671
SLC9A2	kahalide f	0.274192	0.034004
TBP	Fludarabine	0.273587	0.034417
SERPINB5	Imexon	-0.27323	0.034659
TBP	Imexon	0.27189	0.035595
CA2	Sonidegib	0.270702	0.036441
KCTD14	Cisplatin	-0.27044	0.036632
KCTD14	Homoharringtonine	-0.27043	0.036638
SLC9A2	Dabrafenib	-0.27024	0.036778
SERPINB5	Sunitinib	0.269543	0.037281
SLC9A2	Hypothemycin	-0.26954	0.037287
CA2	Lapatinib	0.268928	0.037734

HABP2	Megestrol acetate	0.267873	0.038521
KCTD14	Floxuridine	-0.26733	0.038934
TBP	Chlorambucil	0.2668	0.039335
PI3	1st Precursor Intermediate to TDP 665759	0.266645	0.039454
CD3G	Clofarabine	0.265849	0.040069
TBP	Amonafide	0.265777	0.040124
SERPINB5	Temsirolimus	-0.26558	0.040274
TREML2	Digoxin	0.265228	0.040553
STC2	Idelalisib	0.264339	0.041256
SLC9A2	ABT-199	-0.26407	0.041469
TREML2	Dromostanolone Propionate	0.264026	0.041506
TBP	Uracil mustard	0.263125	0.042232
CD3G	Mitoxantrone	0.263051	0.042292
TREML2	Crizotinib	0.262945	0.042378
TREML2	Nitrogen mustard	0.262709	0.042571
TRIM27	Cytarabine	0.26256	0.042692
SERPINB5	Pazopanib	-0.26247	0.042764
TREML2	Eribulin mesilate	0.262088	0.043081
PI3	Batracylin	-0.26184	0.043286
SERPINB5	Dolastatin 10	0.261554	0.043523
SLAMF7	Idarubicin	-0.26153	0.043543
TREML2	Erlotinib	-0.26126	0.04377
CD3G	kahalide f	-0.26125	0.043777
KCTD14	7-Tert-butyldimethylsilyl-10-hydroxycamptothecin	-0.26064	0.044288
PI3	Bafetinib	-0.26052	0.044394
TRIM27	Imexon	0.259888	0.044927
STC2	Amonafide	-0.25962	0.045155
SLAMF7	Topotecan	-0.25959	0.045181
CA2	Etoposide	-0.25942	0.045329
SERPINB5	Topotecan	-0.2592	0.045518
STC2	Methotrexate	-0.259	0.045688
TBP	Oxaliplatin	0.258941	0.045742
TRIM27	Carmustine	0.258446	0.046172
CD3G	Trametinib	-0.25833	0.046274
CA2	AZD-9291	0.257817	0.046724
TREML2	Pemetrexed	0.257758	0.046776
TRIM27	Doxorubicin	-0.25769	0.046837
TBP	Pemetrexed	0.257457	0.047043
SLC9A2	Bortezomib	-0.25726	0.047215
SERPINB5	Bleomycin	-0.25659	0.047817
CD3G	LY-294002	-0.2565	0.047897
SLAMF7	Trametinib	0.256144	0.048219
KCTD14	Everolimus	-0.25592	0.048424
TRIM27	Curcumin	0.25581	0.048521

SERPINB5	Everolimus	-0.25578	0.048546
CD3G	Simvastatin	-0.25574	0.048582
TBP	Dasatinib	-0.25563	0.048689
CD3G	Dacarbazine	0.255188	0.04909
TRIM27	Chlorambucil	0.255138	0.049136
TRIM27	Dimethylaminoparthenolide	0.255055	0.049212
SLC9A2	Clofarabine	-0.25488	0.049376
PI3	Uracil mustard	-0.25473	0.049508
SLAMF7	Floxuridine	-0.25441	0.049806
CA2	geldanamycin analog	-0.25429	0.049919
TBP	Cyclophosphamide	0.254214	0.049991

Table S6. Pearson correlation between drug IC50 and gene expression.
